# Engineering the poly(A) tail for therapeutic mRNA: from expression control to manufacturing robustness

**DOI:** 10.3389/fbioe.2026.1838589

**Published:** 2026-05-13

**Authors:** Yang-Yang Zhang, Jian-Ping Zhang, Xiao-Bing Zhang

**Affiliations:** 1 State Key Laboratory of Experimental Hematology, National Clinical Research Center for Blood Diseases, Haihe Laboratory of Cell Ecosystem, Institute of Hematology & Blood Diseases Hospital, Chinese Academy of Medical Sciences & Peking Union Medical College, Tianjin, China; 2 Tianjin Institutes of Health Science, Tianjin, China

**Keywords:** dsRNA impurities, in vitro transcription, innate immunity, manufacturability, mRNA therapeutics, poly(A) tail, quality by design, sequence engineering

## Abstract

Messenger RNA (mRNA) therapeutics have advanced rapidly, yet sequence design must now satisfy not only biological potency but also scalable manufacturing, product consistency, and immune compatibility. The 3′poly(A) tail is increasingly recognized as a pivotal factor linking these competing demands. While long homopolymeric tails can enhance translation and prolong RNA persistence, their repetitive nature can also compromise DNA template stability, increase *in vitro* transcriptional slippage and 3′-end heterogeneity, and promote the formation of immunostimulatory byproducts. We define this design trade-off as the Production–Expression paradox, in which sequence features that favor expression may simultaneously undermine manufacturability and immune safety. In this review, we summarize the molecular functions of the poly(A) tail in regulating translation and mRNA decay, and discuss emerging engineering strategies that move beyond conventional linear homopolymers, including segmented designs, chemical modifications, and experimental topological engineering through structured 3′modules, along with their potential impacts on protein yield (often termed a “translational tax”). We further connect upstream production-associated defects to downstream innate immune activation and outline a proposed Quality-by-Design framework for poly(A) optimization, linking critical quality attributes with fit-for-purpose analytical methods ranging from routine release assays to nucleotide-resolution profiling. Reframing poly(A) architecture as an engineerable design parameter, rather than a fixed default element, may improve product comparability, reduce immunogenic burden, and support the more robust translation of mRNA medicines into clinical applications.

## Graphical Abstract

**Figure:**
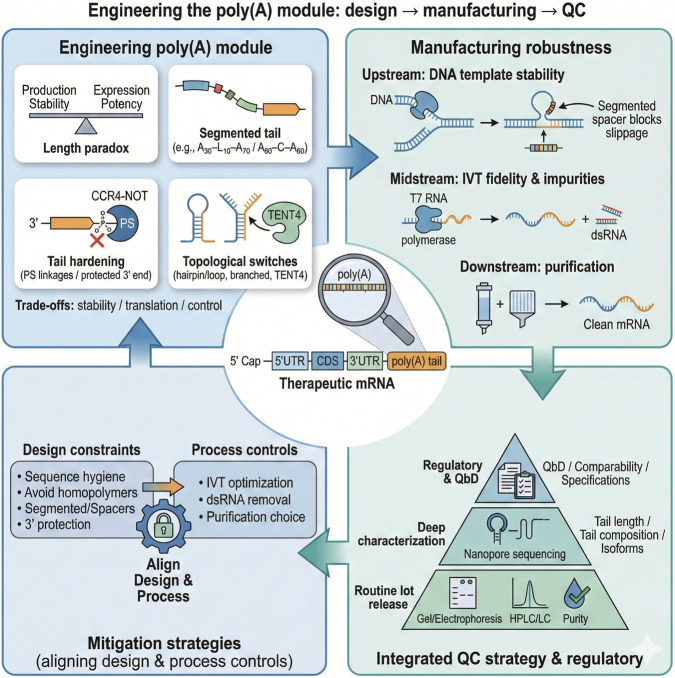

## Introduction

Messenger RNA (mRNA) therapeutics have revolutionized modern medicine, a journey that began with the first proof-of-concept of *in vivo* expressed mRNA and culminated in the rapid deployment of COVID-19 vaccines (Wolff et al., 1990; Baden et al., 2021; Polack et al., 2020; Sahin et al., 2014). Beyond infectious diseases, this modality is now expanding into protein replacement therapies and cancer immunotherapies (Sahin et al., 2014; Holtkamp et al., 2006). A major determinant of the pharmacokinetic profile and therapeutic efficacy of these molecules is the 3′polyadenosine (poly(A)) tail (To and Cho, 2021), a homopolymeric sequence typically ranging from 100 to 250 nucleotides in mammalian transcripts (Edmonds et al., 1971). While historically viewed as a monotonous, passive appendage protecting the 3′end, the poly(A) tail is now increasingly recognized as a versatile engineering module subject to precise optimization (Sahin et al., 2014). Mechanistically, it functions as a scaffold for Poly(A)-Binding Proteins (PABP) to facilitate the “closed-loop” conformation with the 5′cap, thereby governing the delicate kinetic balance between translation initiation and mRNA decay—a process often described as a “molecular timer” for intracellular protein output (Passmore and Coller, 2022; Gallie, 1991; Biziaev et al., 2024; Svitkin et al., 2009; Kahvejian et al., 2001).

However, the engineering of therapeutic poly(A) tails faces what we describe here as a fundamental “Production-Expression Paradox.” Biologically, longer tails (typically >100 nt) are generally required to maximize translational potency and extend half-life in mammalian cells (Passmore and Coller, 2022; Biziaev et al., 2024). Conversely, in the context of manufacturing, long homopolymeric tracts can induce instability in bacterial plasmid templates, leading to recombination, deletion, and polymerase slippage (Macdonald et al., 1993; Molodtsov et al., 2014; Gholamalipour et al., 2018). This conflict forces developers to navigate a narrow design space where biological optimality may clash with manufacturing robustness. Consequently, the poly(A) tail is emerging as a focal point for Design for Manufacturing (DFM), potentially determining not only the drug’s efficacy but also the consistency and safety of the final drug substance (Sahin et al., 2014; To and Cho, 2021).

In this Review, we treat the poly(A) tail as a primary engineering variable in therapeutic mRNA development. Rather than discussing tail properties only in descriptive terms, we organize the field around the logistical and biological decisions developers face: which tail architecture to choose, which risk each design is intended to mitigate, which new liabilities it may introduce, and which analytical controls are required to verify performance. We first summarize the well-established mechanistic basis by which poly(A) tails regulate translation and decay. We then examine emerging design strategies that move beyond conventional linear homopolymers, evaluating how these architectures reshape the trade-off between potency and production robustness. Next, we discuss how template- and IVT-derived failure modes propagate into impurity formation and innate immune sensing, and how process controls and purification strategies can reduce these risks. We then place poly(A) characterization within a proposed Quality by Design (QbD) framework, emphasizing the transition from approximate sizing methods to release-compatible and sequence-resolved analytical workflows (Hornung et al., 2006; Kato et al., 2006; Pichlmair et al., 2006; Kawai and Akira, 2010). Finally, we extend this discussion to non-canonical RNA platforms, including circular RNA, where initial poly(A)-mimetic or poly(A)-inspired strategies are beginning to serve analogous translational functions.

### Scope and literature selection

This review is intended as a narrative review rather than a systematic review. The literature discussed here was selected to support three interconnected aims: (i) to summarize the mechanistic basis by which poly(A) tails influence translation and mRNA decay; (ii) to examine how poly(A)-related design choices affect manufacturability, impurity formation, and analytical control in therapeutic mRNA development; and (iii) to highlight emerging architectures and design concepts that may extend or reconfigure poly(A)-associated functions. Priority was given to studies that were mechanistically informative, experimentally well characterized, or directly relevant to development-stage decision-making in RNA therapeutics and CMC. Foundational studies were included where necessary to establish biological principles, whereas more recent reports were used to illustrate evolving engineering strategies, analytical methods, and translational considerations. Because the field is developing rapidly and includes areas with uneven levels of validation, some sections are intended to be illustrative rather than exhaustive, particularly those addressing emerging topological architectures, AI-assisted design, and forward-looking translational frameworks.

## Biological basis of poly(A) tail regulation

**FIGURE 1:**
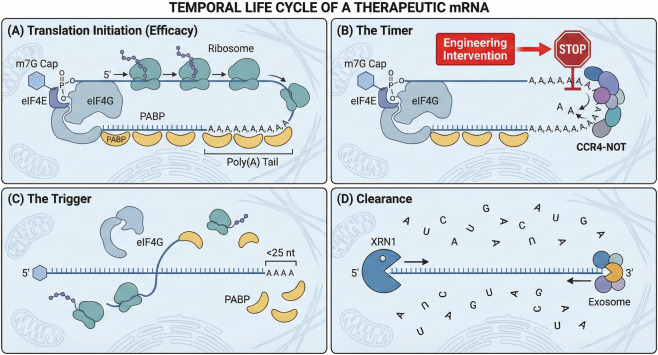
Core regulatory functions of the poly(A) tail in translation and mRNA decay. **(A)** Schematic overview of cap–tail communication in cytoplasmic mRNA translation. The 5′cap and 3′poly(A) tail are shown as functionally linked through translation initiation factors and poly(A)-binding proteins (PABP), illustrating a widely used closed-loop framework for understanding translational control. **(B)** Simplified view of progressive deadenylation, highlighting PAN2–PAN3 and CCR4–NOT as the major deadenylase systems acting on the poly**(A)** tail. **(C)** Conceptual representation of reduced PABP occupancy as tail length shortens, with loss of cap–tail coupling and reduced translational support. **(D)** Downstream consequences of advanced deadenylation, including decapping and exonucleolytic decay. This figure is intended as a mechanistic synthesis of well-supported concepts and does not imply that all steps occur with identical kinetics in every cellular context.

Poly(A) tail engineering can be framed as a stoichiometry-and-kinetics problem: PABP occupancy is a major driver of translation, while deadenylation kinetics largely determine the time-to-decay (Figure 1).

### The PABP footprint and closed-loop formation

Although *in vivo* mRNA conformations appear highly dynamic rather than rigidly “circular,” (Gallie, 1991; Miao and Hu, 2025; Tarun and Sachs, 1996) the closed-loop model remains a highly useful framework for therapeutic mRNA. Cytoplasmic poly(A)-binding protein 1 (PABPC1) is the key mediator of this cap–tail synergy (Nicholson-Shaw et al., 2022). PABP binds the tail with an ∼27 nt footprint (Baer and Kornberg, 1980), and recruits eIF4G to stabilize cap-bound initiation complexes, which in turn is thought to enhance productive translation (Gallie, 1991; Nicholson-Shaw et al., 2022; Re et al., 2014; Vicens et al., 2018; Jackson et al., 2010). Consequently, a minimal tail of ∼20–30 nt is considered the threshold to support the first functional PABP binding event (To and Cho, 2021; Xiang and Bartel, 2021). For therapeutic use, tails commonly exceed ∼100 nt to enable cooperative PABP occupancy, improve ribosome recycling, and sterically protect the 3′end from nucleolytic access (Dever and Green, 2012; Wells et al., 1998; Munroe and Jacobson, 1990; Safaee et al., 2012).

### Deadenylation kinetics and mRNA stability

mRNA durability is extensively regulated by deadenylation, executed in a biphasic manner by PAN2–PAN3 and CCR4–NOT (Yamashita et al., 2005; Collart and Panasenko, 2012; Wahle and Winkler, 2013). When tail length falls below ∼25 nt, PABP dissociates, exposing the transcript to decapping (DCP2) and 3′→5′degradation (exosome), thereby committing the mRNA to rapid clearance (Passmore and Coller, 2022; Garneau et al., 2007). Importantly, CCR4–NOT is highly processive on uninterrupted poly(A); non-A residues can stall its catalytic core. While spontaneous 3′uridylation (e.g., via TUT4/7) accelerates decay (Lim et al., 2014), engineered G/C interruptions have been shown to impede CCR4–NOT processivity, offering a potential strategy to extend the functional window without simply “buffering” by length (Chang et al., 2014; Lee et al., 2024; Lim et al., 2018).

### The coupling between translation and decay

Translation and decay exist in a competitive relationship through shared access to the poly(A) tail (Webster et al., 2018; Coller and Parker, 2005). High PABP occupancy promotes initiation while sterically limiting deadenylase engagement, coupling active translation to increased stability (Chang et al., 2014; Bartel, 2018; Collart, 2016). Conversely, deadenylation erodes the PABP–eIF4G bridge, reducing protein output and licensing decay (Preiss et al., 1998; Torkzaban et al., 2025). These mechanistic principles motivate architectures that preserve sufficient PABP occupancy while slowing CCR4–NOT—design constraints that intersect directly with manufacturability.

## Poly(A) tail engineering: from linear sequences to structured designs

**FIGURE 2:**
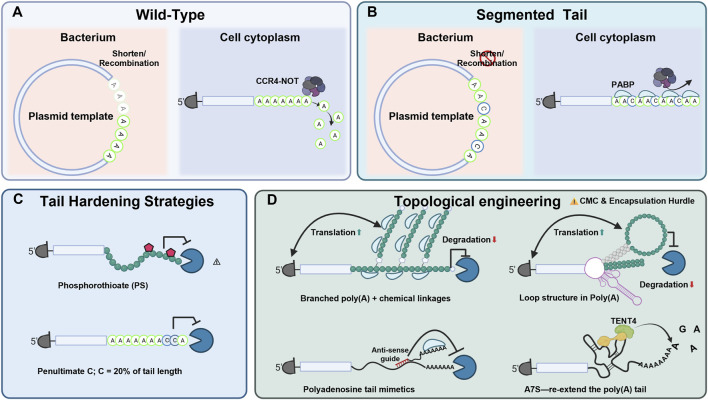
Representative engineering strategies for modifying the poly(A) region of therapeutic mRNA. **(A)** Conventional long homopolymeric poly(A) tails, which may support translation and persistence but can also present upstream template-stability challenges during DNA-based manufacturing. **(B)** Segmented architectures in which non-A spacers interrupt homopolymeric continuity, a strategy reported to improve template stability while maintaining or, in some systems, enhancing RNA performance. **(C)** Tail-hardening approaches, including terminal chemical modification and non-A residue incorporation, which are being explored to reduce deadenylation or prolong functional persistence. **(D)** Emerging structured or topological 3′designs, including looped, branched, or repair-associated motifs, which may alter RNA stability or translational behavior but currently remain less mature from a development and CMC standpoint.

Harnessing the biological principles outlined above, the poly(A) tail can be reconceptualized. Rather than being viewed merely as a static structural appendage, it can serve as a potential “programmable timer” that helps orchestrate the kinetic equilibrium between translational output and mRNA decay (Passmore and Coller, 2022; Nicholson and Pasquinelli, 2019). While endogenous tails are dynamic substrates subject to constant enzymatic remodeling (Collart, 2016; Nicholson and Pasquinelli, 2019), therapeutic mRNA typically demands engineered stability to withstand the rigors of manufacturing and sustain *in vivo* potency (Rosa et al., 2021). Current strategies have evolved from simple length optimization to segmentation, chemical “armoring,” and proposed 3D topological engineering (Figure 2).

### The length paradox: production stability vs expression potency

Therapeutic mRNA design faces what has been termed the “Production-Expression Paradox”: while high-level expression in many mammalian systems generally requires long tails, E. coli—the standard production host for plasmid templates—often exhibits intrinsic instability toward long homopolymeric tracts (Trepotec et al., 2019).

*The Biological Demand:* In eukaryotic cells, poly(A) tail length often correlates positively with translational output, driven by the cooperative binding of PABP (Gallie, 1991; Wells et al., 1998). Recent high-resolution analyses in specific cell-free systems suggest that while mRNA stability increases linearly with tail length, translational initiation efficiency actually peaks at approximately 75 nt and may decline beyond 100 nt; studies suggest that this is possibly because longer tails provide diminishing translational benefit and may impose structural constraints (Biziaev et al., 2024; Peng and Schoenberg, 2005).

*The Manufacturing Ceiling:* Conversely, bacterial manufacturing imposes strict physical and thermodynamic constraints. Long poly (dA:dT) sequences (>100 bp) in plasmids are widely regarded as hotspots for polymerase slippage and homologous recombination (Bzymek and Lovett, 2001). These repetitive tracts have been shown to induce the formation of non-B DNA structures (e.g., triplexes or bent DNA), which stall the replication fork and trigger extensive deletions, leading to heterogeneity in the final mRNA drug substance (Trepotec et al., 2019; Grier et al., 2016).

*Practical Compromise Length:* Across platforms, many developers target an initial tail length on the order of ∼80–120 nt as a starting point (Holtkamp et al., 2006), then tune length in the context of the full construct (5′/3′UTRs, ORF, nucleoside chemistry, cap structure) and the intended dose and route (To and Cho, 2021; Linares-Fernández et al., 2020; Cai et al., 2021; Kim et al., 2024). In this range, PABP occupancy is thought to support closed-loop translation, while limiting some of the known manufacturing liabilities. Importantly, the optimal length appears to be context-dependent and is typically justified using potency, durability, and innate immune readouts together with lot-to-lot distribution control (Linares-Fernández et al., 2020).

### Segmented design for improved template stability

To address the ∼100 nt plasmid bottleneck and achieve more durable expression, segmented poly(A) tails have emerged as a practical engineering strategy. By inserting short non-adenosine linkers, these designs disrupt the homopolymeric continuity of the DNA template (Trepotec et al., 2019). This modification can substantially reduce bacterial recombination associated with long homopolymeric tracts without compromising the biological function of the RNA (To and Cho, 2021).

*The Spacer Evolution:* Early successful designs, such as Pfizer/BioNTech’s BNT162b2 (A30-L10-A70), utilized a 10-nt linker to stabilize the template (European Medicines Agency EMA, 2021). Trepotec et al. (2019) demonstrated that splitting a 120 nt tail into two 60-nt segments (A60-L-A60) drastically reduced plasmid recombination loss from ∼50% to negligible levels (Trepotec et al., 2019).

*Spacer Composition and Design Trade-offs:* The sequence of the linker also matters. While guanosine (G) spacers are superior for stabilizing DNA templates compared to C or T (Trepotec et al., 2019), they introduce a trade-off: G-rich RNA sequences can form G-quadruplexes or trigger innate immune sensors, potentially compromising cell viability (Li C. Y. et al., 2022). Therefore, the choice of spacer should be viewed as a balance between upstream plasmid stability (favoring G) and downstream immunogenicity (favoring C- or A-rich linkers).

*Hyper-segmentation:* Recent studies have extended this concept further. Spiewla et al. (2026) introduced a hyper-segmented design using repeating CA15 motifs (Spiewla et al., 2026). This architecture may provide dual benefits: the repetitive insertion of cytidines (C) disrupts homopolymeric continuity, thereby improving plasmid stability, and may also slow deadenylation by introducing distributed interruptions within the tail. This design stabilized tails exceeding 200 nt and reportedly provided a substantial increase in protein yield compared to standard A90 tails (Spiewla et al., 2026). These findings suggest that segmented architectures may enable the use of tail lengths that are otherwise difficult to manufacture reliably.

### Tail hardening strategies to resist deadenylases

Since deadenylation by the CCR4–NOT complex is the rate-limiting step of decay, protecting the 3′terminus is a potentially effective strategy for extending mRNA half-life (Webster et al., 2018; Collart, 2016).

*Sequence Doping:* Leveraging the substrate specificity of deadenylases, researchers have incorporated non-adenosine residues—specifically cytidines (C)—at the 3′end. These C residues may act as local barriers to nuclease progression, delaying the onset of decay (Li C. Y. et al., 2022).

*Chemical Modification:* A more robust approach involves chemical alteration of the backbone. Introducing phosphorothioate (PS) linkages at the 3′end provides increased resistance to exonucleases while maintaining translational activity (Strzelecka et al., 2020). This strategy is conceptually related to stabilization approaches long used in antisense oligonucleotides (ASOs) (Crooke et al., 2021). However, directly transferring ASO-type modifications to much larger therapeutic mRNAs (∼2,000 nucleotides) creates two major development challenges. These span both formulation and safety: on the formulation side, hydrophobic PS linkages may alter the tail’s charge and flexibility, disrupting mRNA–lipid interactions and yielding suboptimal LNP morphology (An et al., 2022; Cullis and Hope, 2017); on the safety side, extensive PS incorporation has been associated with liver injury, platelet activation/thrombocytopenia, and off-target protein binding, making non-human primate (NHP) validation, beyond standard murine models, essential for robust translational assessment (Frazier, 2015; Valenzuela et al., 2023).

### Topological engineering of structured 3′modules: benefits and trade-offs

Poly(A) engineering is extending from linear sequence design to 3′topological architectures that resist decay, but these gains must be weighed against added CMC and *in vivo* delivery complexity.

*Structural Shielding and the Manufacturing Constraints:* Structured 3′modules, including looped termini (Oh et al., 2025) and branched poly(A) tails (Chen et al., 2025), can reduce exonucleolytic access and increase local PABP density, but they usually require post-transcriptional assembly, lowering GMP yield, increasing batch variability, and complicating characterization. Their altered size and rigidity may also affect LNP encapsulation and endosomal escape, while structured 3′ends may increase the risk of unintended dsRNA-like motifs that engage RIG-I/MDA5 (Schlee et al., 2009). Consequently, robust implementation requires rigorous quality control (QC) gates, such as advanced biophysical characterization (e.g., SEC-MALS) combined with highly sensitive innate immune profiling.

*Dynamic Repair and Pharmacokinetic Variability:* Biomimetic motifs such as A7S can recruit TENT4A/B to restore tail length in the cytoplasm, prolonging mRNA persistence (Lim et al., 2018; Kim et al., 2020; Jung et al., 2025). However, this mechanism depends on host TENT4 abundance, creating cell type–and tissue state–dependent pharmacokinetics (GTEx Consortium, 2020). Sustained competition for TENT4 may also perturb endogenous RNA homeostasis under repeated dosing (Lim et al., 2018; Hyrina et al., 2019). To adequately manage these risks, analytical strategies must shift toward cell-line-specific potency and half-life assays tailored to the intended clinical target tissue.

*Regulatory Switches and Co-delivery Hurdles:* Tail-linked control systems, including ASO-recruited poly(A) mimetics (Torkzaban et al., 2025) and 5′UTR pA regulators (Luo et al., 2024), enable inducible translation but impose demanding co-delivery requirements. Precise co-delivery of mRNA and a second regulator is difficult *in vivo* and often results in discordant biodistribution and reduced efficacy (Geary et al., 2015; Li S. et al., 2022; Hou et al., 2021). Validating such multi-component systems therefore necessitates advanced QC gates, leveraging dual-fluorophore tracking in NHP or complex animal models to accurately map *in vivo* co-delivery kinetics.

### A comparative framework for poly(A) design in therapeutic development

The transition from wild-type poly(A) homopolymers to engineered topologies reframes tail selection as a multi-parameter optimization problem, since maximizing *in vitro* stability yields diminishing returns if it inadvertently exacerbates *in vivo* immunogenicity or undermines GMP feasibility.

To support development decisions, we constructed a benchmarking matrix (Table 1) comparing poly(A) architectures across plasmid stability, expression kinetics, GMP scalability, immunogenicity risk, and the Intellectual Property (IP)/Freedom to Operate (FTO) constraints.

**TABLE 1:** Interpretive comparison of representative poly(A) engineering strategies for therapeutic mRNA development.

Poly(A) architecture	Design logic	Template stability/plasmid compatibility	Reported expression/persistence effect	Manufacturing feasibility	Immune-related concerns	IP/FTO considerations	Evidence basis
Conventional linear WT tails (>100 nt)	Long uninterrupted poly(A) tail	Often limited	Strong in some settings; length-dependent	Simple concept; upstream burden at long length	Generally low; impurity risk indirect	Relatively open	Well supported
Segmented tails	Non-A spacers interrupt homopolymer	Often improved	Maintained or improved; system-dependent	High practicality	Spacer-dependent	Increasingly crowded	Supported by multiple studies
3′end chemical hardening	Terminal protection against deadenylation	Limited upstream benefit	Potentially improved persistence	Moderate complexity	Mixed; added chemistry-related liabilities	Chemistry-dependent	Context-dependent
Structured/topological 3′modules	Looped, branched, or structured tails	Not primarily template-focused	Potentially improved persistence	Often limited	Possible dsRNA-like features	Design-specific	Evidence emerging
Dynamic repair motifs (e.g., A7S/TENT4-related)	Recruit host machinery to maintain tail function	Compatible if sequence is stable	Potentially prolonged persistence	Biologically variable	PK and homeostasis concerns	Mechanism-specific	Promising but early
Tail-linked regulatory/co-delivery systems	Translation control by co-delivered regulator	Depends on encoded backbone	Inducible, but delivery-dependent	Low to moderate practicality	Multi-component liability	Potentially constrained	Exploratory

This table provides an interpretive comparison of representative poly(A) engineering strategies intended to support conceptual evaluation in therapeutic mRNA, development. The categories summarize general design logic, commonly discussed advantages, and frequently noted liabilities in the literature, but they should not be interpreted as a standardized performance ranking or meta-analytic conclusion. The “Evidence basis” column is included to distinguish more mature areas from emerging or exploratory concepts.

As the matrix highlights, linear WT tails >100 nt are associated with substantial manufacturing liabilities, with plasmid recombination rates reaching ∼50% in standard cloning hosts. Segmented tails (e.g., A60–L–A60 or hyper-segmented CA repeats) currently offer a favorable balance of low recombination, strong translational performance, and GMP-compatible manufacturability, although their IP landscape is becoming increasingly crowded (Trepotec et al., 2019; Spiewla et al., 2026). By contrast, branched or looped architectures provide stronger biological shielding but face major industrial constraints: they rely on post-transcriptional chemical coupling or complex folding, which caps process yield and raises CMC characterization and regulatory burden (Oh et al., 2025; Chen et al., 2025). Ultimately, the optimal poly(A) design is modality-dependent: acute indications (e.g., vaccines) favor robust manufacturability (segmented tails), whereas chronic replacement therapies may justify higher CMC complexity for self-regenerating or topologically hardened modules.

## Manufacturing robustness: overcoming poly(A) hurdles

Transitioning poly(A) design from the bench to industrial scale demands a robust Design for Manufacturing (DFM) framework. The core challenge lies in the intrinsic thermodynamic instability of long homopolymers, which manifests differently across distinct production phases—from plasmid propagation to enzymatic synthesis. To ensure batch-to-batch consistency and regulatory compliance, developers should deploy a multi-tiered strategy encompassing host engineering, strict process control, and purification logic (Table 2).

**TABLE 2:** Representative failure modes and control strategies for poly(A)-related mRNA manufacturing.

Process stage	Representative failure mode	Likely root cause	Potential impact	Representative mitigation	Suggested detection/control	Evidence basis
Plasmid propagation	Tail deletion or contraction	Replication slippage; recombination	Template heterogeneity; altered tail length	Segmented tails; strain selection	Plasmid sequencing; encoded-tail verification	Well supported
Plasmid maintenance/fermentation	Plasmid multimerization	Recombination; segregational instability	Lower effective yield; higher COGs	Circular architectures; multimer-control systems	Topology assessment; yield monitoring	Supported
Template architecture selection	Poor manufacturability of specialized vectors	Linear or low-productivity vector formats	Reduced scalability	Favor simpler encoded designs when possible	Template qualification; COG comparison	Context-dependent
IVT reaction	Tail-length heterogeneity	T7 slippage on homopolymers	Broad tail distribution; batch inconsistency	Reduce repetitiveness; optimize IVT conditions	Tail-distribution analysis	Well supported
IVT reaction	dsRNA byproduct formation	Self-templating; antisense synthesis; IVT kinetics	Innate immune activation; lower tolerability	Process tuning; downstream dsRNA removal	dsRNA assays; functional immune testing	Well supported
Polymerase/reagent sourcing	Supply-chain fragility	Reliance on specialized enzymes	Cost and GMP risk	Prefer broadly available reagents when possible	Vendor qualification; comparability testing	Development-oriented
Downstream purification	Residual truncated/tailless transcripts	Abortive transcription; incomplete separation	Heterogeneity; immunostimulatory species	Oligo-dT enrichment; orthogonal cleanup	Transcript sizing; impurity profiling	Supported
Downstream purification	Residual dsRNA contamination	Incomplete impurity clearance	RIG-I/MDA5 activation risk	Cellulose purification; RP-HPLC	dsRNA-specific assays	Well supported
Tail installation strategy	Poor comparability with enzymatic tailing	Broad stochastic tail-length distribution	Weaker lot control	Prefer template-encoded tails for controlled products	CGE; sequence-resolved methods	Supported
Sequence design/linker composition	Unintended immune liability	U-rich motifs; GC-rich structures; dsRNA-like pairing	Increased innate sensing risk	Lower-risk linker design; construct-level validation	Structure review; immune testing	Evidence emerging
Integrated control strategy	Over-reliance on one assay	Multi-stage defect sources	Incomplete QC; weak root-cause analysis	Tiered control strategy	CGE + sequencing + functional assays	Strong practical support

This table summarizes representative poly(A)-related manufacturing risks and corresponding control strategies across different process stages. It is designed as a practical, fit-for-purpose framework rather than a complete inventory of all failure modes, mitigations, or analytical controls. The examples shown may vary in relevance depending on construct architecture, manufacturing platform, purification workflow, and intended clinical application.

### Upstream controls: stabilizing the DNA template

The primary source of manufacturing failure occurs before transcription begins: the instability of the plasmid DNA template. While segmented tails (discussed previously) are the primary DFM solution to disrupt homopolymer continuity (Trepotec et al., 2019; Li C. Y. et al., 2022), residual recombination risks in long poly (dA:dT) tracts persist, necessitating further intervention (Molodtsov et al., 2014; Triana-Alonso et al., 1995; Friehs and Scheper, 2004).

*Host Engineering:* Standard cloning strains often fail to maintain long repetitive sequences. Consequently, the use of specialized *Escherichia coli* strains (e.g., Stbl3 or recA-deficient derivatives) is often advantageous. Crucially, simple recA deficiency is often insufficient, as long homopolymers remain susceptible to RecA-independent recombination instability mechanisms (e.g., replication slippage) (Bzymek and Lovett, 2001; Al-Allaf et al., 2013). Therefore, strains should be specifically validated to suppress these residual instability mechanisms, preserving the integrity of full-length templates during bacterial propagation.

*Combating Segregational Instability:* A more subtle issue is the formation of plasmid multimers (Field and Summers, 2011). Poly(A) tracts can accelerate homologous recombination between plasmid copies, driving the formation of dimers, trimers, and higher-order multimers (Friehs and Scheper, 2004; Field and Summers, 2011). This reduces the number of independent segregation units and can lead to uneven partitioning during cell division, thereby increasing plasmid loss during fermentation (Field and Summers, 2011; Viguera et al., 2001). To counteract this, modern therapeutic vectors may incorporate active resolution systems, such as cer sites (which utilize the host XerCD recombinase to resolve multimers back into monomers) or active partitioning loci (e.g., parABS), to support stable inheritance (Bzymek and Lovett, 2001; Field and Summers, 2011).

*Vector Architecture and the Cost-of-goods Trade-offs:* To propagate extreme tail lengths (∼500 nt) that exceed the practical limits of circular vectors, linear vectors such as pEVL (derived from pJazz) use telomere-capped ends to bypass recombination entirely (Grier et al., 2016). However, this approach introduces substantial manufacturing bottlenecks. Poor *E. coli* fermentation titers increase cost of goods (COGs) relative to standard high-copy circular vectors, while the altered physiology of the required host strains can complicate downstream alkaline lysis and primary recovery. Consequently, linear plasmids may remain too costly for broad deployment, favoring scalable segmented tails within standard circular architectures in many manufacturing settings (Trepotec et al., 2019; Grier et al., 2016).

### Midstream controls: IVT fidelity and impurity mitigation

Even with a stable DNA template, the *in vitro* transcription (IVT) reaction introduces its own fidelity issues. T7 RNA polymerase is prone to transcriptional slippage on homopolymers, in which the enzyme loses register on the template, resulting in tail-length heterogeneity (±5%–10%) and premature termination (Macdonald et al., 1993; Molodtsov et al., 2014; Triana-Alonso et al., 1995).

*Polymerase Engineering and Supply Chain Constraints:* Emerging strategies focus on engineering T7 polymerase variants with improved register fidelity. Analogous to the development of high-fidelity polymerases for next-generation sequencing (NGS) (Arslan et al., 2024; Leconte et al., 2010), these variants could in principle reduce indel errors and slippage. However, their industrial implementation currently faces major supply-chain barriers (Kuzmine and Martin, 2001; Wu et al., 2021; Jiang et al., 2001). In contrast, wild-type (WT) T7 is already a widely available, industry-standard GMP reagent. Switching to custom variants may create dependence on a limited supplier base and substantially increase costs. Until multiple vendors can reliably provide these enzymes, WT T7 is likely to remain the more practical option for many manufacturing workflows.

**FIGURE 3:**
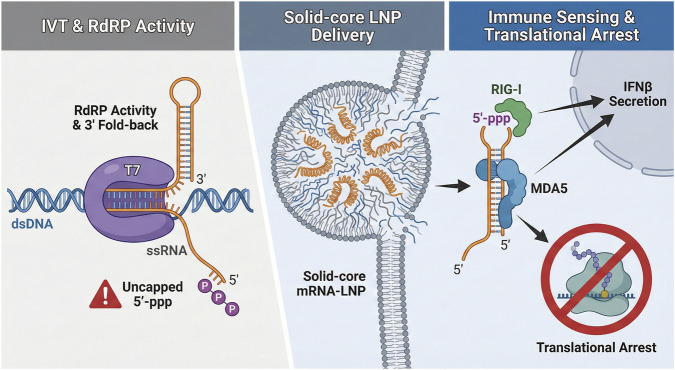
Proposed links between poly(A)-associated IVT defects, dsRNA impurity formation, and innate immune activation. Left: During *in vitro* transcription, homopolymeric or structurally permissive RNA regions may contribute to polymerase slippage, 3′-end heterogeneity, or self-templated extension events, which have been associated with formation of dsRNA-like byproducts in some IVT systems. Middle: These byproducts may persist into downstream formulation unless effectively removed by purification, thereby coexisting with the intended therapeutic RNA cargo. Right: Upon cellular delivery, such aberrant RNA species may engage innate immune sensors, including pathways associated with RIG-I-like receptors, PKR, and OAS/RNase L, with potential downstream effects on RNA stability and translation.

*Suppressing dsRNA:* At the same time, reliance on WT T7 requires manufacturers to manage its known byproducts. One of the most important safety concerns at this stage is the formation of double-stranded RNA (dsRNA). The poly(A) tail may serve as a hotspot for RNA-dependent RNA polymerase-like activity, in which T7 polymerase engages the 3′end of the nascent transcript to synthesize a complementary antisense strand (Figure 3) (Gholamalipour et al., 2018; Mu et al., 2018). Process tuning can help suppress this mechanism (i) Thermodynamic Control: High-temperature IVT (e.g., 42 °C): destabilizes the transient RNA:DNA hybrids that promote slippage (Wu et al., 2020). (ii) Kinetic Control: A “low steady-state UTP feeding” strategy creates a kinetic bottleneck that stalls backward-slipping polymerases, effectively reducing dsRNA byproducts by 60%–70% (Ziegenhals et al., 2023). (iii) Additives: The inclusion of chaotropic agents can further disrupt tail self-pairing, maintaining the RNA in a single-stranded state (Piao et al., 2022).

### Downstream controls: purification and strategy selection

The final quality gate involves removing truncated species and making strategic choices regarding tail-addition methods.

*Purification Strategy:* For the mRNA product, oligo-dT affinity chromatography remains the industry standard for enriching full-length tails and separating them from abortive transcripts (Baiersdörfer et al., 2019; Holtkamp et al., 2006). However, affinity purification alone is insufficient to address all safety concerns. Removal of dsRNA contaminants—often by RP-HPLC or cellulose purification—remains an important step in limiting innate immune activation, consistent with the principle established by Karikó et al. (Baiersdörfer et al., 2019; Karikó et al., 2011)

*Template-encoded* versus *Enzymatic Tailing:* Finally, manufacturers must choose between encoding the tail in the template and adding it enzymatically. While enzymatic tailing offers flexibility during early-stage research, it produces a stochastic distribution of tail lengths. In a Quality by Design (QbD) context, template-encoded polyadenylation is strongly preferred for GMP manufacturing because it provides better reproducibility and tighter length control. Despite the upstream cloning challenges, this approach is better aligned with the need to maintain tail length as a controlled quality attribute across production batches (Rosa et al., 2021; Pardi and Krammer, 2024).

## Mitigation strategies: aligning sequence design and process control

To reduce innate immune recognition that can compromise therapeutic efficacy, manufacturers should employ an integrated strategy. This approach combines process purification (to remove immunostimulatory byproducts) with structural design (to minimize immunogenic features at the sequence level) (Table 2).

### Purification: beyond IVT optimization

While upstream optimizations of the *in vitro* transcription (IVT) reaction—such as high-temperature synthesis (Wu et al., 2020) and low-UTP feeding (Ziegenhals et al., 2023)—significantly reduce the baseline formation of double-stranded RNA (dsRNA), they rarely eliminate it entirely. Consequently, downstream purification remains an important phase for improving product safety.

*dsRNA Depletion:* The removal of dsRNA contaminants is a high priority, as even trace amounts can trigger RIG-I and MDA5 sensors (Hornung et al., 2006; Kato et al., 2006; Pichlmair et al., 2006; Kawai and Akira, 2010; Mu et al., 2018; Alexopoulou et al., 2001). Cellulose chromatography has emerged as a scalable and robust method for selectively depleting these byproducts (Baiersdörfer et al., 2019). High-capacity affinity chromatography has also been reported to enable scalable removal of dsRNA impurities from IVT mRNA (Clark et al., 2025). Alternatively, RP-HPLC remains a widely used high-resolution method for separating dsRNA based on hydrophobicity, building on purification principles established in earlier work by Karikó et al. (Karikó et al., 2011)

*Enzymatic Treatment:* Some processes employ RNase III, an enzyme specific to double-stranded RNA, to digest helical regions in the crude product. However, this approach requires rigorous control to prevent unwanted cleavage of therapeutically relevant RNA structures (Baiersdörfer et al., 2019).

*Integrity Control:* Finally, oligo-dT affinity purification acts as an important quality-control step (Holtkamp et al., 2006; Baiersdörfer et al., 2019). By selecting for full-length poly(A) tails, this step also reduces the abundance of tailless abortive transcripts. These truncated species may be particularly immunostimulatory because their exposed 5′-triphosphates can serve as ligands for RIG-I activation (Hornung et al., 2006; Pichlmair et al., 2006; Schlee et al., 2009; Baiersdörfer et al., 2019).

### Sequence design: structural considerations and linker constraints

A complementary strategy is to reduce immunogenicity through sequence design itself. Beyond widely used chemical modifications (e.g., N1-methylpseudouridine) (Baden et al., 2021; Karikó et al., 2005), the structural properties of the poly(A) region may also influence innate immune recognition.

*Homopolymeric* versus *Structured Tails:* Pure homopolymeric poly(A) sequences are often favored because they tend to remain flexible and predominantly single stranded, enabling efficient PABP binding. In contrast, more complex or GC-rich tail sequences may form intramolecular hairpins that resemble dsRNA-like structures and could increase innate immune sensing (Mu et al., 2018).

*Linker composition in segmented tails:* When segmented tails are used to improve plasmid stability, linker composition becomes an important design variable. G/C-containing spacers may be preferable to U-rich linkers in some contexts (Trepotec et al., 2019). U-rich regions may introduce motifs recognized by TLR8 or facilitate non-specific antisense hybridization, thereby promoting dsRNA formation (Kawai and Akira, 2010; Li C. Y. et al., 2022; Diebold et al., 2004; Heil et al., 2004).

### Poly(A) integrity and immune exposure

Taken together, these observations suggest that the poly(A) tail contributes not only to mRNA stability and translation, but also to the extent to which the transcript remains exposed to innate immune surveillance.

In a properly engineered therapeutic mRNA, a full-length tail recruits PABP, supports closed-loop formation, and promotes active translation. This PABP-associated state may also reduce immune exposure by limiting access of some sensors to the 3′end and by favoring engagement of the transcript with the translation machinery rather than with cytosolic surveillance pathways (Collart, 2016; Luo et al., 2024).

Conversely, tail truncation can erode PABP protection, reduce translational efficiency, and increase the likelihood that transcripts enter stress-associated RNA states. Under these conditions, the RNA may become more exposed to pathways involving Protein Kinase R (PKR) and 2′-5′-Oligoadenylate Synthetase/RNase L, with downstream consequences including eIF2α phosphorylation and translational arrest (Mu and Hur, 2023). In this sense, maintaining poly(A) integrity is not only a manufacturing objective, but also a biologically relevant determinant of intracellular RNA behavior.

## Integrated QC strategy and regulatory context

**FIGURE 4:**
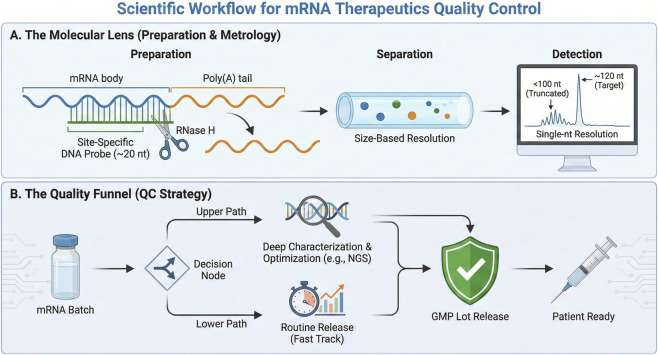
Tiered analytical framework for poly(A)-tail characterization in therapeutic mRNA quality control. **(A)** Probe-guided workflow for poly(A)-tail measurement. Site-specific probe annealing and RNase H cleavage are used to generate a poly(A)-containing fragment, which is subsequently analyzed by size-based separation and high-resolution detection. **(B)** Fit-for-purpose QC strategy for mRNA batch assessment. Rapid and scalable assays may support routine release testing and lot-to-lot distribution control, whereas higher-content methods, such as sequencing-based approaches, may be reserved for process development, troubleshooting, and comparability studies. The framework illustrates how analytical methods can be selected according to the required level of resolution and the specific quality decision being made.

In practice, a one-size-fits-all analytical strategy is insufficient. Instead, a tiered quality-control (QC) framework is more appropriate, distinguishing between high-throughput methods for routine manufacturing and high-content methods for deeper biological and structural characterization (Figure 4) (Rosa et al., 2021).

### Routine lot release: pragmatic assays for distribution control

For routine GMP manufacturing, capillary gel electrophoresis (CGE) serves as a practical quantitative assay (Di Grandi et al., 2023; Wu et al., 2024; Webb et al., 2025). Its adoption is driven by its speed, precision, and operational similarity to established protein QC methods (e.g., CE-SDS), making it accessible to many QC laboratories. CGE provides a rapid profile of tail-length distribution and can support batch-level consistency assessment when aligned with predefined specifications (Table 3) (United States Pharmacopeia USP, 2023).

**TABLE 3:** Fit-for-purpose comparison of representative methods for poly(A)-tail analysis.

Analytical technique	Typical output	Resolution	Turnaround time	Main limitations	Best use context	Evidence basis
CGE	Tail-length distribution	High	Hours	No direct sequence information; sensitive to sample design	Routine QC; lot comparison; release-supporting analysis	Well supported
IP-RP HPLC	Bulk size/purity profile	Low to moderate	Hours	Limited precision for tail-length assignment	Orthogonal purity check; in-process control	Established, but limited for precise tail sizing
3′AIM-seq/related short-read NGS	Tail composition; non-A residues; sequence-level variation	High	Days	Less robust for very long homopolymers; higher workflow complexity	Development; method validation; root-cause analysis	Strong in development settings
Direct RNA sequencing (ONT)	Full-length RNA context; isoforms; approximate tail length	Moderate	Hours to days	Limited precision for exact homopolymer length	Exploratory characterization; full-length transcript analysis	Useful for context, less suitable for release quantification
RT-ddPCR	Targeted abundance or threshold readout	Not length-resolving	Hours	Does not measure tail-length distribution directly	Rapid process check; targeted monitoring	Useful as a supportive assay

This table compares representative analytical methods that may be used to assess poly(A)-tail attributes in different settings, including development, investigation, comparability, and routine control. The entries are intended to highlight typical strengths, limitations, and use contexts, rather than to imply universal equivalence or a fixed regulatory hierarchy. Method suitability remains context-dependent and should be justified according to the specific product, question, and stage of development.

### Deep characterization: sequence-resolved methods for development and comparability

However, physical sizing alone cannot resolve sequence-level defects. During process characterization, method development, or root-cause investigation, next-generation sequencing (NGS)-based methods—such as 3′AIM-seq—or long-read platforms such as Oxford Nanopore Technologies (ONT) can provide higher-resolution information on tail composition and sequence fidelity. These methods may help identify untemplated additions, non-A residues, or other sequence features that are not readily captured by electrophoretic sizing alone (Table 3) (Workman et al., 2019; Subtelny et al., 2014; Krause et al., 2019; Begik et al., 2023; Legnini et al., 2019).

### Regulatory interpretation and QbD alignment

From a Quality by Design (QbD) perspective, it is increasingly reasonable to treat poly(A)-tail attributes as quality-relevant analytical features when they are linked to product performance, consistency, or comparability. Draft and emerging guidance documents, including those cited here, support closer attention to RNA structural and sequence-related attributes, but the extent to which poly(A)-tail properties are formally designated or assessed may vary across regulatory context, product class, and stage of development.

Accordingly, manufacturers should be prepared to justify whether tail length, composition, and distribution should be monitored as part of an overall control strategy, particularly when these features may influence potency, stability, or immunogenicity. In comparability settings, it may also be important to show that poly(A)-related attributes remain acceptably consistent with the clinical reference material used in pivotal studies (European Medicines Agency EMA, 2021; European Medicines Agency, 2025; U.S. Food and Drug Administration FDA, 2022; Abraham et al., 2010).

Consequently, the use of at least one validated quantitative method for poly(A)-tail distribution analysis may be justified within a release or comparability framework, while higher-resolution assays may be reserved for development, investigation, or extended characterization (Table 3). With robust metrology established, the next step is to integrate these measurements into a broader QbD framework for process understanding and control.

## Poly(A)-inspired design in emerging RNA architectures

As RNA therapeutics evolve beyond linear formats, the functions traditionally associated with the poly(A) tail are increasingly being re-examined in other architectural contexts. Rather than viewing the poly(A) tail solely as a terminal appendage, recent studies suggest that some of its key roles—particularly PABP recruitment and translational support—may, in certain settings, be partially recapitulated in alternative topologies. By functionally separating PABP recruitment from the physical position of a 3′end, these approaches expand current design strategies for RNA stability and translation.

### Circular RNA and internal poly(A)-based translational support

Circular RNAs (circRNAs) present an important design contrast to linear mRNAs: their covalently closed structure confers strong resistance to exonucleases (Enuka et al., 2016), whereas the absence of a 5′cap and 3′tail has historically limited translational efficiency relative to linear mRNA (Legnini et al., 2017). To improve their translational performance, recent biomimetic strategies have attempted to recreate some features of cap–tail functional coupling within a circular scaffold (Chen et al., 2023).

*Mechanistic Basis:* Early studies showed that eukaryotic ribosomes could engage circular templates containing internal poly(A) tracts (Chen and Sarnow, 1995). More recent engineering efforts have extended this concept by introducing a precisely spaced internal poly(A) segment (typically ∼12–20 nt) to recruit PABP within the circular RNA (Chen et al., 2023). When combined with an internal ribosome entry site (IRES) and an aptamer capable of recruiting eIF4G, this configuration may help functionally bridge translation-supporting elements that, in linear mRNA, are normally coordinated through cap–tail communication (Abe et al., 2015; Tong et al., 2025; Shen et al., 2025; Khan et al., 2023). In some reported systems, such architectures have approached the protein output of linear mRNAs (Abe et al., 2015; Tong et al., 2025; Shen et al., 2025; Khan et al., 2023).

*Design Constraints in Circular Architectures:* However, these architectures also introduce important design constraints. Internal poly(A) elements require careful placement, because ribosomal read-through into inappropriate sequence contexts may promote poly-lysine-associated stalling or frameshifting (Juszkiewicz and Hegde, 2017). In addition, the cyclization process can leave structured junctional sequences that may alter translation behavior or increase the likelihood of dsRNA-like features (Pichlmair et al., 2006; Schlee et al., 2009). If such junctions are translated aberrantly, they could in principle generate unintended peptide products, which may be relevant when considering repeated dosing or chronic applications. Analytically, these considerations support the use of junction-resolved RNA characterization to confirm the integrity of the internal PABP-binding region after ligation (Ma et al., 2019). From a translational perspective, the immunogenicity and *in vivo* tolerability of these engineered junctions would likely require careful evaluation in advanced preclinical models.

### Structural analogues and topological hybrids

Beyond circular RNAs, other emerging architectures use structured elements to substitute for, mimic, or stabilize functions typically associated with the poly(A) tail.

*Viral-derived Structural Motifs in SaRNA:* Alphavirus-derived self-amplifying RNAs (saRNAs) contain 3′conserved sequence elements (CSEs) that fold into defined secondary structures such as pseudoknots. These elements may slow tail remodeling or degradation by limiting deadenylase access, thereby contributing to transcript persistence in some vaccine-associated settings (Khan et al., 2023; Erasmus et al., 2020).

*Dumbbell RNAs:* A related concept is represented by dumbbell RNA architectures (Abe et al., 2015). By sealing both termini with tight stem-loops, these molecules achieve strong resistance to exonuclease attack while retaining a single-stranded poly(A)-containing region for PABP interaction. Conceptually, this design combines enhanced structural stability with retention of a translation-supportive element, although the extent to which these advantages translate across delivery and therapeutic settings remains to be determined (Abe et al., 2015).

*Formulation and Translational Constraints:* A major open question for topological hybrids such as dumbbell RNAs is whether their structural advantages can be maintained through clinically relevant delivery workflows. From a biophysical modeling perspective, it is hypothesized that their rigid double-stranded termini may increase the hydrodynamic radius of the payload relative to flexible linear transcripts. This theoretical alteration in geometry could affect encapsulation behavior in LNP systems originally optimized for linear mRNAs (Li S. et al., 2022; Schober et al., 2024). This altered geometry may also influence internal lipid organization and, potentially, endosomal escape, although the extent of these effects is likely to be formulation-dependent (Carrasco et al., 2021). In addition, encouraging durability observed in mice may not translate directly across species, given known differences in innate immune sensitivity and RNA sensing thresholds (Schlee et al., 2009; Lemdani et al., 2024; Hellgren et al., 2024; Corbett et al., 2021). Accordingly, the comparative translational value of these architectures relative to optimized segmented tails remains unresolved and will require further validation in more predictive preclinical models.

## Discussion

### Architectural evolution and translational CMC

Resolving the Production–Expression paradox will likely require moving beyond simple homopolymeric tails toward segmented and, in some cases, more structurally complex architectures (Trepotec et al., 2019). However, any such shift must be evaluated in the context of manufacturability, analytical controllability, and therapeutic use case. To improve translational feasibility, these design changes may need to be accompanied by a more adaptable Chemistry, Manufacturing, and Controls (CMC) framework (Qin et al., 2022; Pardi et al., 2018). Upstream, this includes layered control of plasmid template integrity before transcription. Downstream, it supports a tiered analytical strategy in which capillary gel electrophoresis (CGE) is used for routine distribution analysis and next-generation sequencing (NGS) or related high-resolution methods are applied for deeper structural characterization when needed. Rather than serving as an absolute guarantee of transcript integrity, this framework is better viewed as a practical basis for process understanding, comparability, and control within a QbD-oriented workflow (International Council for Harmonisation of Technical Requirements for Pharmaceuticals for Human Use ICH, 2009).

### In silico modeling and AI-assisted design

Given the growing complexity of tail architectures and the associated CMC constraints, empirical optimization alone may become increasingly inefficient. In this context, artificial intelligence (AI) and computational biophysics are emerging not as absolute replacements for experimental validation, but as powerful complementary tools for rational design. Beyond optimizing coding sequences for translation and RNA structure (Zhang et al., 2023; Wayment-Steele et al., 2021), future predictive frameworks may also need to model the 3′UTR and poly(A) region as functionally coupled design variables, particularly when sequence context influences stability, immunogenicity, or translational performance (Wayment-Steele et al., 2022; Castillo-Hair et al., 2024; Mo et al., 2025). Likewise, molecular simulations may help evaluate how RNA topology influences LNP formulation behavior before experimental testing, although such approaches are still developing and should be interpreted alongside empirical validation (Carrasco et al., 2021).

### A fit-for-purpose framework for clinical deployment

Ultimately, CMC strategy and computational design should support clinical performance rather than operate as stand-alone optimization goals. From this perspective, it is unlikely that a single poly(A) architecture will be optimal across all therapeutic settings (Qin et al., 2022). A more useful framework may be to align tail design with the intended pharmacodynamic window and treatment context (Pardi et al., 2018; Verbeke et al., 2022). For example, prophylactic vaccines—which typically necessitate acute antigen bursts—may benefit from architectures (such as segmented tails) that balance strong short-term expression with highly scalable manufacturability, whereas other applications may prioritize duration, controllability, or reduced immunostimulatory burden (Pardi et al., 2018). In precision genetic interventions such as CRISPR- or CAR-T-related workflows, prolonged RNA persistence may introduce additional safety or off-target considerations, although the magnitude of such risk will depend on construct design, dosing, and delivery context (Guo et al., 2023; Ewaisha and Anderson, 2023). By contrast, chronic systemic therapies may, in some cases, justify the additional complexity of more durable or regenerative tail architectures if their functional benefits clearly outweigh manufacturing and formulation costs (Jung et al., 2025).

### Concluding perspective

Taken together, current evidence supports the view that poly(A) design should be treated as an adjustable engineering parameter rather than a fixed default feature. Integrating CMC considerations, higher-resolution analytics, emerging computational design tools, and therapeutic context may improve how poly(A) architectures are selected and evaluated across mRNA platforms. In that sense, the poly(A) tail is best understood not as a passive appendage, but as a highly engineerable control module that fundamentally bridges expression kinetics, manufacturing robustness, and therapeutic safety in next-generation RNA medicines (Carrasco et al., 2021; Pardi et al., 2018; International Council for Harmonisation of Technical Requirements for Pharmaceuticals for Human Use ICH, 2009).
